# Expression and clinical significance of CA125, CA153 and CEA in nipple discharge of breast cancer patients

**DOI:** 10.5937/jomb0-45192

**Published:** 2024-04-23

**Authors:** Jun Geng, Shi Jinli, Weina Guo, Haiyan Li, Yang Dan, Yan Gao

**Affiliations:** 1 Jinan Central Hospital, Medical Laboratory Diagnosis Center, Jinan, China; 2 Affiliated Qingdao Central Hospital of Qingdao University, Qingdao Cancer Hospital, Emergency Center, Qingdao, China; 3 Affiliated Qingdao Central Hospital of Qingdao University, Qingdao Cancer Hospital, Health Management Center, Qingdao, China; 4 Zhangqiu District People's Hospital, Department of Anesthesiology, Jinan, China; 5 Zhangqiu District People's Hospital, Department of Otolaryngology, Jinan, China; 6 Dongguan Town Hospital, Medical Laboratory Diagnosis Center, Department of Clinical Laboratory, Dongguan, China

**Keywords:** breast cancer, CA125, CA153, CEA, nipple discharge, rak dojke, CA125, CA153, CEA, iscedak iz bradavica

## Abstract

**Background:**

It is an important clinical means to identify benign and malignant breast diseases caused by nipple discharge through the detection and analysis of components in nipple discharge. This study was aimed to test the expression and clinical significance of carbohydrate antigen 125 (CA125), carbohydrate antigen 153 (CA153) and carcinoembryonic antigen (CEA) in nipple discharge of breast cancer patients.

**Methods:**

From January 2017 to December 2018, 86 patients with invasive ductal carcinoma of the breast with nipple discharge (breast cancer group) and 50 patients with ordinary breast duct hyperplasia with nipple discharge (benign control group) were selected, and the levels of CA125, CA153 and CEA in nipple discharge and serum were detected by electrochemiluminescence immunoassay.

## Abstract (sr)

**Uvod:**

To je važno kliničko sredstvo za identifikaciju benignih i malignih oboljenja dojke uzrokovanih iscedkom iz bradavica kroz detekciju i analizu komponenti u sekretu iz bradavica. Ova studija je imala za cilj da ispita ekspresiju i klinički značaj antigena ugljenih hidrata 125 (CA125), antigena ugljenih hidrata 153 (CA153) i karcinoembrionalnog antigena (CEA) u iscedu iz bradavice kod pacijenata sa karcinomom dojke.

**Metode:**

Od januara 2017. do decembra 2018. odabrano je 86 pacijenata sa invazivnim duktalnim karcinomom dojke sa iscedkom iz bradavice (grupa karcinoma dojke) i 50 pacijenata sa običnom hiperplazijom kanala dojke sa iscedkom iz bradavice (benigna kontrolna grupa), a nivoi CA125, CA153 i CEA u sekretu iz bradavica i serumu detektovani su elektrohemiluminiscentnim imunotestom.

**Rezultati:**

Nivoi CA125, CA153 i CEA u sekretu iz bradavice i serumu u benignoj kontrolnoj grupi bili su značajno niži od onih u grupi sa karcinomom dojke, a nivoi CA125, CA153 i CEA u serumu su očigledno bili nž'i od onih u sekretu iz bradavica. Nivoi ekspresije CA125, CA153 i CEA u sekretu iz bradavica u grupi sa karcinomom dojke nisu imali značajnu razliku u različitim godinama početka i različitim mestima tumora. Nivoi CA125, CA153 i CEA u sekretu iz bradavice kod pacijenata sa prečnikom tumora 5 cm, niskom diferencijacijom, visokim stadijumom, metastazama i recidivom su očigledno bili povišeni u odnosu na one kod pacijenata sa prečnikom tumora <5 cm, visokom diferencijacijom, niskim stadijumom, i bez metastaza i recidiva. Ovi nivoi nisu bili u značajnoj korelaciji sa ekspresijom receptora estrogena (ER) i receptora progesterona (PR), ali su bili značajno povezani sa ekspresijom receptora humanog epidermalnog faktora rasta (HER-2) i Ki-67. Tačnost, osetljivost i negativna prediktivna vrednost kombinovane detekcije CA125, CA153 i CEA u sekretu iz bradavica u dijagnozi raka dojke su značajno poboljšane u poređenju sa kombinovanom detekcijom seruma i pojedinačnim otkrivanjem.

**Zaključak:**

Kombinacija CA125, CA153 i CEA u sekretu iz bradavica može se smatrati potencijalnom dijagnostičkom metodom za karcinom dojke, koja je efikasan dodatak serološkoj dijagnozi i može dati nove ideje za diferencijalnu dijagnozu benignog i malignog raka dojke sa iscedak iz bradavica.

## Introduction

Breast cancer is a tumor occurring in the breast area. It has become the most common malignant tumor in women. The incidence of breast cancer in developed countries in Europe and the United States has reached 100/100000, and the incidence in large and medium-sized cities in China is 50–60/100000 [1]. Recently, the breast cancer incidence in China has been on the rise, and the early age of onset has attracted attention. Therefore, strengthening the differential diagnosis of benign and malignant breast diseases is of great significance for the early diagnosis and treatment of breast cancer to improve its prognosis and reduce the mortality rate. Nipple discharge is a clinical manifestation of some breast diseases. It is a pathological discharge spontaneously secreted by breast ducts. Differential diagnosis of breast diseases through the detection of components in nipple discharge is a potential differential diagnosis method [2]. The traditional cytological examination of nipple discharge exfoliation is used to diagnose breast cancer, but its diagnostic sensitivity is low. The tumor molecular markers are used as tumor-related substances secreted directly by tumor cells to detect whether their expressions in nipple discharge can be improved. The accuracy and sensitivity of breast cancer diagnosis are worth exploring. Therefore, this study intends to detect the CA125, CA153 and CEA levels in nipple discharge, analyze the relationship with breast diseases, and explore the feasibility of detecting nipple discharge tumor markers in breast cancer diagnosis, which provides new ideas for breast cancer diagnosis and treatment.

## Materials and Methods

### Clinical data

A total of 86 patients with invasive ductal carcinoma of the breast with nipple discharge (breast cancer group) were collected, including 44 patients with early breast cancer (stage I +II) and 42 patients with middle and advanced breast cancer (stage III +IV). 50 patients with ordinary breast duct hyperplasia with nipple discharge were selected as benign control group. Breast cancer group: both genders were female, aged 31–72 years, mean (53.21±10.02) years old. Benign control group: both genders were females, aged 30–71 years old, mean (52.64±11.35) years old, patients in both groups were diagnosed by histopathology. Inclusion criteria for the breast cancer group: (1) The diagnosis met the WHO diagnostic criteria [3]; (2) Unilateral nipple discharge; (3) The data required for the study were complete, including test data, age of onset, tumor location, tumor size, degree of differentiation, and Tumor Node Metastasis (TNM) staging, tumor recurrence and metastasis and ER, PR, HER-2 and nuclear proliferation index Ki-67, etc.; (4) They did not receive anti-tumor therapy and endocrine therapy before treatment. Exclusion criteria: (1) Exclude patients with other systemic malignant tumors; (2) Patients with incomplete clinical data required for the study; (3) Patients with pituitary prolactin adenoma and other tumors causing milk secretion. There was no statistical difference in age between the two groups of subjects, and they were comparable. The study was approved by the ethics committee of Jinan Central Hospital (approval no. 2017-01-0089) and conducted with the consent of the patients, who signed the informed consent form.

### Methods

#### Specimen collection

Nipple discharge collection: Before collection, wipe the patient’s nipple with a 75% medical alcohol cotton ball to remove dirt and cell debris on the nipple surface; manually squeeze the affected breast from the periphery of the breast to the nipple in the direction of the duct. The nipple discharge was collected in sterile Eppendorf tubes, and the supernatant was collected by centrifugation and stored at 4℃ until use.

#### Serum Specimen Collection

From 6: 00 to 8: 00 in the morning, 3 mL of cubital venous blood was drawn from the patients on an empty stomach.

#### Inspection method

The inspection method of CA125, CA153 and CEA is electrochemiluminescence, the principle is double antibody sandwich principle, the instrument is Roche ELecsys-2010, and the reagent is Roche original reagent (Basel, Switzerland).

#### Immunohistochemical examination methods

**Figure 1:**
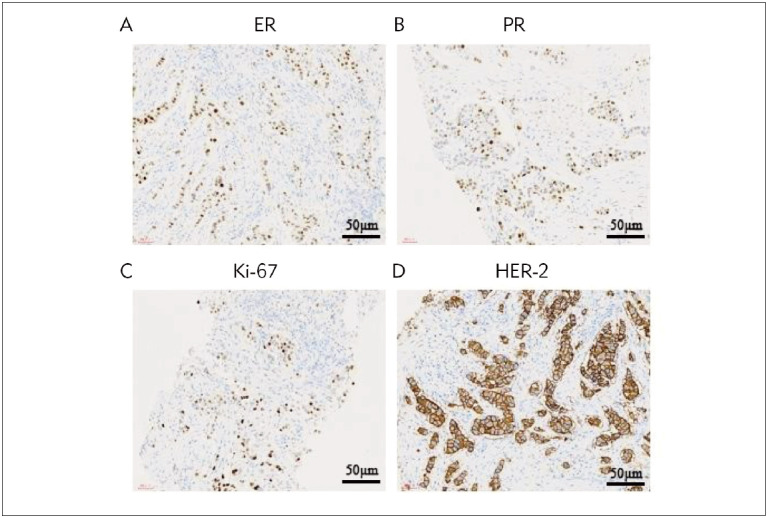
ER, PR, and Ki-67, and HER-2 in mastectomy tissue were detected by Immunohistochemical Ultra Sensitive TM S-P method. (A): ER (70% positive cells). (B): PR (65% positive cells). (C): Ki-67 (40% positive cells). (D): HER-2 (+++).

HER-2, PR, ER, and Ki-67 in mastectomy tissue were detected by Immunohistochemical Ultra Sensitive TM S-P method. Routine 3-4 um sections, the sections were dewaxed and embedded with standard procedures, positive and negative controls were set, and the immunoreactive signal was displayed with DAB substrate. HER-2 cell membranes are positive, and more than 10% of cancer cells expressing HER-2 in a tumor are considered HER-2 positive. Additionally, for PR, ER, and Ki-67 nuclear positivity, the percentage of nuclear positive cancer cells was recorded. Tumor ER and PR positive expression rate > 10% was evaluated as positive, and tumor Ki-67 positive expression rate > 14% was evaluated as positive. All these were counted at 200× and the average dada were selected, as shown in Figure 1A, Figure 1B, Figure 1C, Figure 1D.

#### Statistical methods

SPSS25.0 statistical software (IBM, NY, USA) was used for data analysis and processing. All the data were tested for normality by Kolmogorov- Smirnov and Shapiro-Wilk. Normally distributed data are expressed as mean ± standard deviation. Student’s t-test was used for comparison between two groups. Categorical variables were presented as frequency (percentage) (n (%)), and comparison of two groups was performed using a chi-squared test. One‑ways ANOVA was used for comparison among the multiple groups. Spearman was applied for correlation analysis. ROC curve was drawn to obtain the truncation values of CEA, CA153 and CA125 of nipple discharge in the diagnosis of breast cancer. With pathological diagnosis as the gold standard, the value of combined detection of CEA, CA153 and CA125 in nipple discharge in the diagnosis of breast cancer was statistically calculated by four grid table method. P<0.05 is considered as statistically significant.

## Results

### Measurement of nipple discharge and serum levels of CA125, CA153 and CEA

The nipple discharge levels of CEA, CA153 and CA125 in breast cancer group and early breast group were significantly higher than those in the benign control group (P<0.05) (Table 1). Similarly, the serum levels of CEA, CA153 and CA125 in breast cancer group and early breast group were significantly higher than those in the benign control group as well (P<0.05) (Table 2). Furthermore, the levels of CEA, CA153 and CA125 in breast cancer discharge group were significantly higher than those in breast cancer serum group (P<0.05) (Table 3).

**Table 1:** Comparison of the nipple discharge levels of CEA, CA153, and CA125 in three groups. Note: vs. Benign control discharge group, ^a^P<0.001; vs. Early breast cancer discharge group, ^b^P<0.001

Group	Cases	CEA (ng/mL)	CA153 (U/mL)	CA125 (U/mL)
Benign control discharge group	50	6.28±1.80	26.69±6.13	31.23±8.01
Early breast cancer discharge group	44	29.31±13.12^a^	112.34±39.83^a^	117.52±39.78^a^
Breast cancer discharge group	86	72.83±30.21^a,b^	181.22±57.33^a,b^	196.52±65.12^a,b^
F		158.111	192.449	179.376
P		<0.001	<0.001	<0.001

**Table 2:** Comparison of the serum levels of CEA, CA153, and CA125 in three groups. Note: vs. Benign control serum group, aP<0.001; vs. Early breast cancer serum group, bP<0.001.

Group	Cases	CEA (ng/mL)	CA153 (U/mL)	CA125 (U/mL)
Benign control serum group	50	2.96±0.52	18.01±4.36	22.43±6.43
Early breast cancer serum group	44	3.15±0.64	20.13±5.31	23.64±6.49
Breast cancer serum group	86	45.23±11.14^a,b^	99.62±32.59^a,b^	105.36±40.13^a,b^
F		668.465	279.598	191.626
P		<0.001	<0.001	<0.001

**Table 3:** Comparison of nipple discharge and serum CEA, CA153 and CA125 levels in breast cancer group. Note: vs. Breast cancer serum group, ^a^P<0.001.

Group	Cases	CEA (ng/mL)	CA153 (U/mL)	CA125 (U/mL)
Breast cancer serum group	86	45.23±11.14	99.62±32.59	105.36±40.13
Breast cancer discharge group	86	72.83±30.21^a^	181.22±57.33^a^	196.52±65.12^a^
t		7.949	17.029	11.052
P		<0.001	<0.001	<0.001

### The relationship between CA125, CA153 and CEA levels in nipple discharge and clinicopathological factors in breast cancer group

There was no significant difference in the levels of CA125, CA153 and CEA in the nipple discharge in the breast cancer group in different age of onset and different tumor sites (P>0.05). The expression levels of CEA, CA153, and CA125 in nipple discharge of patients with tumor diameter >5 cm, low differentiation, high stage, metastasis, and recurrence were significantly higher than those of patients with tumor diameter 5 cm, high differentiation, low stage, and no metastasis and recurrence (P<0.05) (Table 4).

**Table 4:** Relationship between CA125, CA153 and CEA levels and clinicopathological factors in nipple discharge in breast cancer group (x̄±s). Note: Comparison within groups: ^a^P<0.05.

Group	Cases <br>(n)	CEA <br>(ng/mL)	t/F	P	CA153 <br>(U/mL)	t/F	P	CA125 <br>(U/mL)	t/F	P
Age (year)			1.953	0.054		1.271	0.207		1.349	0.181
50	39	75.34±30.26			171.26±49.16			186.27±66.04		
>50	47	63.64±25.31			186.24±58.36			205.37±64.79		
Tumor location			1.616	0.109		0.594	0.554		0.643	0.522
left breast	46	64.03±23.97			182.34±54.36			201.36±77.81		
right breast	40	72.83±26.52			175.21±56.81			191.42±63.44		
Tumor diameter (cm)			43.142	<0.001		25.247	<0.001		47.698	<0.001
<2	18	23.16±9.43^a^			101.83±32.56^a^			98.65±36.29^a^		
2–5	30	58.69±16.52			179.42±49.31			169.51±39.28		
>5	38	96.25±39.27			213.67±66.31			265.39±83.62		
Clinical stage			10.724	<0.001		9.319	<0.001		10.230	<0.001
I+II	44	29.31±13.12^a^			112.34±39.83^a^			117.52±39.78^a^		
III+IV	42	109.25±47.63			250.31±89.37			279.31±96.71		
Differentiation			13.261	<0.001		9.274	<0.001		10.977	<0.001
High and medium	59	31.74±13.25^a^			134.22±52.30^a^			139.78±42.37^a^		
Low	27	150.54±66.42			279.23±92.36			322.56±112.20		
Lymph node metastasis			1.970	0.052		2.575	0.012		1.683	0.096
No	35	60.09±16.37^a^			154.32±41.56^a^			171.44±44.29^a^		
Yes	51	69.75±25.61			186.49±65.32			201.37±98.47		
Distant metastasis			14.183	<0.001		8.456	<0.001		8.236	<0.001
No	69	39.46±17.85^a^			149.55±50.34^a^			166.78±55.92^a^		
Yes	17	186.78±79.82			302.45±112.44			321.49±109.44		
Relapse			14.727	<0.001		8.971	<0.001		8.540	<0.001
No	65	35.87±15.69^a^			143.86±52.46^a^			158.43±59.61^a^		
Yes	21	176.33±72.64			296.27±102.14			318.41±109.64		

### Correlation analysis between the levels of CA125, CA153 and CEA in nipple discharge and the HER-2, PR, ER and Ki-67 expression in cancer tissues

The CA125, CA153 and CEA levels in nipple discharge in breast cancer group had no significant correlation with the PR and ER expression, but were significantly correlated with the HER-2 and Ki-67 expression (P<0.05) (Table 5).

**Table 5:** Correlation analysis between the nipple discharge CA125, CA153 and CEA levels and PR, ER, Ki-67 and HER-2 in cancer tissues.

Project	ER	PR	HER-2	Ki-67				
	r	P	r	P	r	P	r	P
CEA	0.140	0.556	0.375	0.103	0.682	0.001	0.581	0.007
CA153	0.376	0.102	0.427	0.060	0.527	0.017	0.823	<0.001
CA125	0.076	0.751	0.275	0.240	0.796	<0.001	0.836	<0.001

### The value of combined detection of the levels of CA125, CA153 and CEA in nipple discharge in the diagnosis of breast cancer

**Figure 2:**
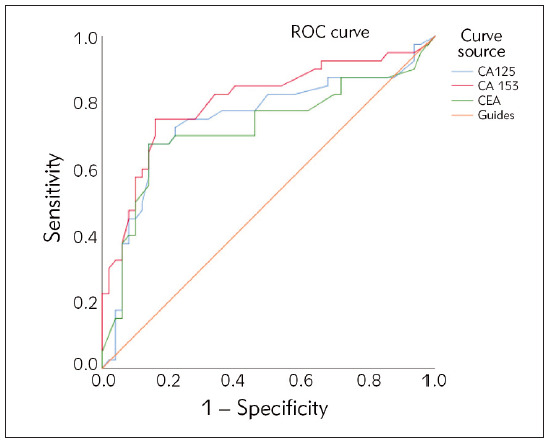
The ROC curve of nipple discharge CEA, CA153 and CA125 in the diagnosis of breast cancer.

The ROC curve was drawn to obtain the cut-off values of CEA, CA153 and CA125 in nipple discharge for diagnosis of breast cancer. The area under the curve (AUC) of nipple discharge CEA, CA153 and CA125 in the diagnosis of breast cancer were 0.679 (95% CI: 0.767–0.893), 0.788 (95% CI: 0.716– 0.861) and 0.771 (95% CI: 0.698–0.844) respectively; Yoden index is 0.678, 0.759 and 0.704 respectively; The cut-off values of CEA, CA153 and CA125 in nipple discharge for diagnosis of breast cancer were 9.80 ng/mL, 35.00 U/mL and 40.00 U/mL, respectively, as shown in Figure 2.

Serum and nipple discharge CA125, CA153 and CEA single and combined detection results of breast cancer diagnosis and pathological diagnosis results were compared, as shown in Table 6. Taking pathological diagnosis as the gold standard, the statistical results showed that the accuracy, sensitivity, and negative predictive value of the combined detection of the nipple discharge CA125, CA153 and CEA in the breast cancer diagnosis were obviously improved compared with the serum combination and the individual detections, which were 93.88%, 98.47%, 96.51%, respectively P<0.01 (Table 7).

**Table 6:** Measurement of serum and nipple discharge CA125, CA153 and CEA single and combined detection results of breast cancer diagnosis and pathological diagnosis results (n).

Project	Laboratory diagnosis (n)	Pathological diagnosis (n)	
		malignant	benign
Serum CEA	malignant	44	3
	benign	42	47
CA153	malignant	48	3
	benign	38	47
CA125	malignant	46	4
	benign	40	46
Joint	malignant	65	5
	benign	21	45
Discharge CEA	malignant	55	2
	benign	31	48
CA153	malignant	60	2
	benign	26	48
CA125	malignant	58	3
	benign	28	47
Discharge	malignant	83	4
	benign	3	46

**Table 7:** Measurement of the value of combined detection of the nipple discharge CA125, CA153 and CEA in the breast cancer diagnosis (% (n)). Note: Three joint detection of discharge vs. Three combined detection of serum and individual detections, ^a^P<0.01.

Index	Sensitivity	Specificity	Accuracy	Positive predictive <br>value	Negative predictive <br>value
Serum CEA	51.16 (44/86)	94.00 (47/50)	69.47 (91/131)	93.62 (44/47)	52.81 (47/89)
Serum CA153	55.81 (48/86)	94.00 (47/50)	72.52 (95/131)	94.12 (48/51)	55.29 (47/85)
Serum CA125	53.49 (46/86)	92.00 (46/50)	70.23 (92/131)	92.00 (46/50)	53.49 (46/86)
Three combined detection <br>of serum	75.58 (65/86)	90.00 (45/50)	83.97 (110/131)	92.86 (65/70)	68.18 (45/66)
Discharge CEA	63.95 (55/86)	96.00 (48/50)	78.63 (103/131)	96.49 (55/57)	60.76 (48/79)
Discharge CA153	69.77 (60/86)	96.00 (48/50)	82.44 (108/131)	96.77 (60/62)	64.86 (48/74)
Discharge CA125	67.44 (58/86)	94.00 (47/50)	80.15 (105/131)	95.08 (58/61)	62.67 (47/75)
Three joint detection of <br>discharge	96.51 (83/86)^a^	92.00 (46/50)	98.47 (129/131)^a^	95.40 (83/87)	93.88 (46/49)^a^
X^2^	58.850	2.468	50.225	2.315	29.994
P	0.001	0.929	0.001	0.940	0.001

## Discussion

Nipple discharge is a mammary gland specific proximal fluid that is naturally secreted by the ductlobular system in the breast of non-lactating adult women [3] [4]. It is rich in proteins, hormones, lipids and carbohydrates, and is generally shed from cell fragments, ducts and lobular epithelium, which can indirectly reflect the breast microenvironment under pathological conditions [5]. The vast majority of breast cancer cases originate from the epithelial cells of the duct-lobular system, so nipple discharge is an ideal source of biomarkers before and after cancer, providing a useful reference for the early screening of breast cancer [6].

CA153 is the product of MUC1 gene, and its high expression is usually associated with colon cancer, breast cancer, ovarian cancer, lung cancer, and pancreatic cancer [7]. CA153 has been a classic marker for the diagnosis of breast cancer, and has higher application value in the preoperative prognosis assessment, postoperative disease monitoring and efficacy evaluation of breast cancer patients [8]. CEA is a glycoprotein produced by normal fetal intestinal tissue and epithelial tumors and involved in cell adhesion. It was first identified as a colon cancer antigen in 1965 [9], and it is overexpressed in a variety of human cancers, such as breast, lung, pancreatic and colorectal cancers. As a broad spectrum marker, it is also one of the important biomarkers for the diagnosis of tumors [10]. CEA not only used as a marker for early diagnosis of breast cancer, but also as an important indicator for therapeutic efficacy evaluation and postoperative monitoring of metastasis and recurrence [11]. CA125 is a tumor carbohydrate antigen with a gene located on human chromosome 19 and a relative molecular weight of 200,000 to 1 million, which mainly exists in cells and has a low level in the blood of healthy human beings. When the body is cancerous, CA125 can be released into the blood and used as a biological marker for the diagnosis of ovarian cancer [12]. Studies have shown that CA125 is also highly expressed in other malignant tumors, such as breast cancer and gastric cancer [13], and is involved in regulating the proliferation, differentiation, invasion and metastasis of tumor cells, and is a commonly used tumor marker to reflect the degree of tumor malignancy and recurrence and metastasis [14].

Nipple discharge acts as a »soaking fluid« for tumor cells. When tumor cells do not break through the basement membrane, the expression of blood markers is not high, but the expression of nipple discharge could be abnormal. Tumor markers in nipple discharge not only appear earlier than in serum, but also have a higher concentration than in serum [15]. Therefore, detection of tumor markers in discharge is conducive to the early diagnosis of breast cancer. This study showed that the levels of CEA, CA153 and CA125 in serum and nipple discharge of breast cancer group were significantly higher than those of benign control group, and the levels of CEA, CA153 and CA125 in nipple discharge of breast cancer group were significantly higher than those of serum levels. The levels of CEA, CA153 and CA125 in nipple discharge were closely related to clinicopathological factors. The levels of CEA, CA153 and CA125 in nipple discharge with the tumor diameter 5cm, high clinical stage, low differentiation and recurrence were significantly higher than those in nipple discharge with the tumor diameter 5 cm, low clinical stage, high differentiation and no recurrence and metastasis, and the prognosis could be evaluated according to their expression levels. This study also showed that the positive rate of HER-2 and Ki-67 was significantly correlated with the levels of CEA, CA153 and CA125 in nipple discharge, which also reflected the malignancy of the tumor from the side. Moreover, the sensitivity, accuracy, negative predictive value of CEA, CA153 and CA125 combined detection in nipple discharge for diagnosis of breast cancer were significantly improved compared with serum combination and individual detection, which is conducive to early diagnosis and early clinical intervention.

There are still shortcomings in this study. The enrolled patients in this study were limited to those who visited our hospital. The study will include a multicenter study to better analyze the association with nipple discharge tumor markers and breast disease.

## Conclusion

In conclusion, the detection of nipple discharge tumor markers CA125, CA153 and CEA provides a new potential diagnostic method for the benign and malignant breast diseases with nipple discharge diagnosis, which is helpful to determine the occurrence and development of breast diseases. It will have broad prospects for development and important clinical significance.

## Dodatak

### Conflict of interest statement

All the authors declare that they have no conflict of interest in this work.
